# Response to ‘Is the case really a SMARCA4‐deficient thoracic sarcoma?’

**DOI:** 10.1111/1759-7714.13751

**Published:** 2020-11-18

**Authors:** Kohichi Takada, Shintaro Sugita, Tadashi Hasegawa

**Affiliations:** ^1^ Department of Medical Oncology Sapporo Medical University School of Medicine Sapporo Japan; ^2^ Department of Surgical Pathology Sapporo Medical University School of Medicine Sapporo Japan

We thank Dr Kunimasa for his interest in our case report and agree that a smoking history plus comprehensive immunohistochemical analyses are indispensable for a diagnosis of SMARCA4‐deficient thoracic sarcoma (SMARCA4‐DTS).^1^, ^2^

In this regard, the patient was a current and heavy smoker of 47‐pack‐years. A computed tomography image had revealed emphysema in line with the patient's history of smoking. A smoking history complicated by emphysema was consistent with previous reports, but the tumor size and sites of metastases were atypical of SMARCA4‐DTS.

Second, the immunohistochemical features of our case were as follows: Tumor cells were positive for vimentin, cytokeratin (AE1/AE3), EMA, CD34, p63, synaptophysin and SOX2, focally positive for TTF1 and NUT, and negative for chromogranin A, S‐100, Claudin‐4 and SMARCA2.^3^ Therefore, we diagnosed the tumor as a SMARCA4‐DTS, with immunohistochemical signatures that were compatible with the current recommendations for its diagnosis.^4^ Additionally, the patient underwent commercial DNA‐based tumor genomic profiling (TGP; FoundationOne CDX). TGP identified the following actionable gene alterations; *SMARCA4* E386*, *SMARCA4* E384*, *FLCN* Q354*, *BRD4* P978_P980del, *TP53* R175H, and amplifications of *CD274* (programmed cell death‐ligand 1: PD‐L1*), PDCD1LG2 (PD‐L2)*, and *JAK2*. The results of TGP overwhelmingly support our diagnosis of a SMARCA4‐deficient tumor that overexpresses PD‐L1.

Our case fits the diagnosis of a SMARCA4‐DTS according to comprehensive immunohistochemical studies, except for overexpressed PD‐L1. Notably, the patient has been alive for over a year following pembrolizumab therapy, unlike patients in previous similar reports. This sarcoma type may be categorized as a subtype of SMARCA4‐DTS in future. However, a diagnostic criterion for SMARCA4‐DTS is urgently required to be established in order to develop therapeutic options. We strongly contend that we should not miss the opportunity to treat patients with SMARCA4‐DTS using immune checkpoint inhibitors.

To this end, we apologize for the error in the original version with regard to SMARCB1 and any confusion this might have caused.

## Disclosure

All authors report no conflicts of interest.
